# The removal of 3-monochloropropane-1,2-diol ester and glycidyl ester from refined-bleached and deodorized palm oil using activated carbon

**DOI:** 10.1039/d1ra00704a

**Published:** 2021-05-05

**Authors:** Elvi Restiawaty, Aulia Maulana, Neng Tresna Umi Culsum, Christian Aslan, Veinardi Suendo, Norikazu Nishiyama, Yogi Wibisono Budhi

**Affiliations:** Research Group of Chemical Engineering Process Design and Development, Faculty of Industrial Technology, Institut Teknologi Bandung Jalan Ganesha 10 Bandung 40132 Indonesia erestiawaty@che.itb.ac.id; Department of Bioenergy Engineering and Chemurgy, Faculty of Industrial Technology, Institut Teknologi Bandung Indonesia; Department of Chemical Engineering, Faculty of Industrial Technology, Institut Teknologi Bandung Bandung Indonesia; Division of Inorganic and Physical Chemistry, Faculty of Mathematics and Natural Sciences, Institut Teknologi Bandung Indonesia; Division of Chemical Engineering, Graduate School of Engineering Science, Osaka University Japan

## Abstract

Palm oil has fulfilled most of the oil needs in the food sector in the world. However, palm oil is indicated to contain small amounts of compounds that are harmful to humans, especially to infants. These toxic contaminants are 3-monochloropropanediol (3-MCPD) esters and glycidyl esters (GE), which are formed during the deodorization of palm oil at high temperatures. This study aims to reduce the 3-MCPD ester concentration in refined, bleached, and deodorized palm oil (RBDPO) through adsorption using activated carbon. The activated carbons were treated with heat and acid-washing using HCl at various concentrations and were characterized. The treatment altered the physicochemical characteristics of the activated carbon (surface area, pore volume, pH_PZC_, and CEC), resulting in the enhancement of its adsorption characteristics (adsorption capacity). The activated carbon treated with 2 N HCl (AC 2 N) was chosen as the proper adsorbent, due to better surface area, better pore volume, highest CEC value, and better positive charge in RBDPO. The 3-MCPD and GE adsorption capacity of AC 2 N was 1.48 mg g^−1^ and 29.68 mg g^−1^, respectively. The adsorption ability of pretreated activated carbon towards 3-MCPD esters was examined in a batch system at various adsorption temperatures. The 3-MCPD ester concentration in RBDPO was successfully reduced by up to 80% at 35 °C using the activated carbon treated with 2 N HCl solution. On the other hand, the activated carbon was able to reduce the other contaminant of GE in RBDPO up to 97% from the initial concentration of GE.

## Abstract 2

The removal of 3-MCPD and GE from RBDPO was done through adsorption using activated carbon. The maximum 3-MCPD and GE removals result in 80% and 97%, respectively.

## Graphical abstract

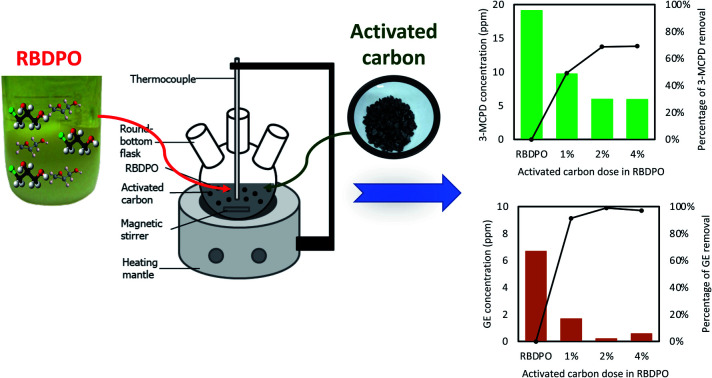

## Introduction

Among all countries worldwide, Indonesia is the highest producer and largest exporter of palm oil. It is estimated that Indonesian palm oil production will reach approximately 38.58 million metric tons in 2021, and it will increase in the coming years. It is also predicted that approximately 24.58 million metric tons of palm oil will be exported to other countries, while the rest (13.99 million metric tons) will be used for domestic consumption.^1^

Palm oil can be processed for production of its derivative products, such as cooking oil and margarine. The process consists of two main steps: extraction and refining. In the extraction step, crude palm oil (CPO) is produced. Then, CPO undergoes the refining step, which consists of physical (steam) refining, degumming, bleaching, and deodorizing to produce refined, bleached, and deodorized palm oil (RBDPO). Next, RBDPO is fractionated to separate the olein and stearin. Finally, the stearin is packed as margarine products, while the olein is packed as cooking oil products.^2^

Every derivative product of palm oil contains 3-monochloropropane-1,2-diol ester (3-MCPDE) and its related substance, glycidyl ester (GE). Both are formed by the deodorizing process at high temperature, which is approximately 200 °C.^3^ Commonly, cooking oil produced from palm oil has the highest 3-MCPDE and GE concentrations at approximately 14 ppm.^4^

3-MCPDE and GE are considered to be harmful to human health because of their capability to induce tumors in rodents. 3-MCPDE is categorized as a nongenotoxic carcinogen, while GE is a genotoxic carcinogen.^5^ Because of this, these compounds must be permitted to enter the human body in the lowest amounts possible. The European Food Safety Authority stated that the maximum concentration of 3-MCPDE and GE that enters the body should be no more than 0.8 μg per kg body weight per day.^3^ According to those recommendations, mitigation steps must be taken to remove those contaminants in palm oil.

There are some mitigation steps for reducing the amount of 3-MCPDE and GE in edible oils, namely, avoidance and minimization of precursors in the raw material, modification, extension of the refining process and removal of the esters after refining.^4^ In this research, removal of the esters after refining with an adsorption method was studied because it has a low cost and does not change the commercial process. The adsorption must be added between the deodorization and fractionation steps (because 3-MCPDE and GE are mostly formed during the deodorization step)^3^ and removed in both saturated and unsaturated fat products. Therefore, RBDPO which was produced after deodorization step and before fractionation step,^2^ was used in this research. Adsorption of 3-MCPDE and GE had been studied by several researchers using many kinds of adsorbents such as zeolite, magnesium silicate, white clay, and activated carbon.^6–8^

Activated carbon is a low-cost adsorbent with excellent adsorption properties. It has been used for adsorption of various chemicals like heavy metal ions,^9,10^ dyes,^11–13^ and organic substances.^14–16^ In previous report, activated carbon has been applied to reduce the concentration of 3-MCPDE and GE.^6,8^ This research shows that activated carbon has a potential as a promising adsorbent for 3-MCPDE and GE removal. However, there is still lack information about the adsorption properties of the activated carbon in 3-MCPDE and GE removals from RBDPO. The adsorption properties studied in this paper include the surface area and pore volume of the adsorbent, the functional groups on the adsorbent surface, the inorganic elements on the adsorbent, pH of zero-point charge, cation-exchange capacity (CEC), and isotherm model for the adsorption of 3-MCPD. Moreover, other research works used untreated palm oil or model solution such as hexadecane solution,^6,7^ while this research used RBDPO as raw material containing 3-MCPDE and GE. Therefore, activated carbon was studied intensively as the adsorbent to remove 3-MCPDE and GE in RBDPO.

## Material and methods

### Materials

RBDPO used in this research was from the palm oil industry in Lampung, Indonesia. Untreated activated carbon was bought from the local industry in Bandung, Indonesia. The 3-monochloropropanediol-d5 (3-MCPD-d5) was used as an internal standard produced from Larodan, Sweden. The chemical reagents used in this experiment were analytical grade and included the following: acetone, ammonium sulfate, chloric acid, ethyl acetate, heptane, methyl *tert*-butyl ether, nitric acid, phenylboronic acid, potassium hydroxide, potassium nitrate, sodium hydroxide, sodium methoxide, and sulfuric acid.

### Adsorbent pretreatment

Activated carbon was used in this research as an adsorbent, which was analyzed by CHN Analyzer (LECO CHN 628, Netherlands), on Chemical Analysis Laboratory, Research Center for Chemistry-Indonesian Institute of Sciences, Serpong, Indonesia. The composition of activated carbon was 75.34 ± 1.27%-w carbon, 2.70 ± 0.12%-w hydrogen, 0.24 ± 0.08%-w nitrogen, and 21.72 ± 1.22%-w oxygen on the dry and sulfur free basis. The activated carbon pretreatment method was heat and acid-wash pretreatment. The activated carbons were heated in an autoclave. The autoclave operating temperature, pressure, and time were 121 °C, 1 bar, and 60 minutes, respectively. The activated carbons were then acid-washed using HCl. The concentration of HCl was varied for 1, 2, and 3 N with a ratio of 10 ml of HCl solution per 1 g of activated carbon. Then, the activated carbon was agitated at room temperature for 24 hours. The activated carbons were then washed with demineralized water until the washing wastewater reached pH 5–7. Finally, the activated carbons were dried in the drying oven. The HCl treated activated carbon samples were then assigned as AC 1 N, AC 2 N, and AC 3 N, respectively.

### Characterization of adsorbents

BELSORP-Max (Microtrac BEL) was used to measure the nitrogen adsorption/desorption at 77 K.^17^ The Brunauer, Emmet, and Teller (BET) method was used to determine the total surface area and the Barrett–Joyner–Halenda (BJH) method was used to determine the pore volume and pore size distribution. The functional groups formed on the surface of activated carbon were detected using Fourier Transform Infra-red Spectroscopy (Prestige21, Shimadzu, Japan). The sample was preparation into pellet and analyzed in 2 cm^−1^ resolution between 4000 and 400 cm^−1^ in a spectral region. Characterization of inorganic elements on activated carbon was carried out using Orbis Micro X-ray fluorescence (XRF) analyzer operated at 40 kV.

### pH of zero-point charge (pH_PZC_) of the adsorbents

The simplified potentiometric mass titration Method^18^ was used to determine pH_PZC_. Blank solution was made by adding 9 ml of 0.1 M KNO_3_ and 18 ml of deionized water into a beaker glass. Then, 3 ml of 0.01 M KOH was added into the solution. The solution was then titrated with 0.01 M HNO_3_ until constant pH was reached. For the sample, 150 mg of adsorbent was added to into the blank solution. Then, 3 ml of 0.01 M KOH was added and titrated with 0.01 HNO_3_ until constant pH was reached. The value of pH_PZC_ was determined from the intersection between the pH plots of the blank solution and the sample.

### Cation exchange capacity of the adsorbents

Cation exchange capacity (CEC) was measured and calculated using a modified version of Boehm's technique.^19,20^ The treated activated carbon (0.1 gram) was placed into a vial bottle. Then, 20 ml of 0.1 N NaOH was added to the vial. The vial was shaken for 24 hours. After that, the solution in the vial was titrated using 0.1 N HCl. After the titration, the NaOH concentration after 24 hours was calculated. The CEC was calculated using eqn (1),^19^1
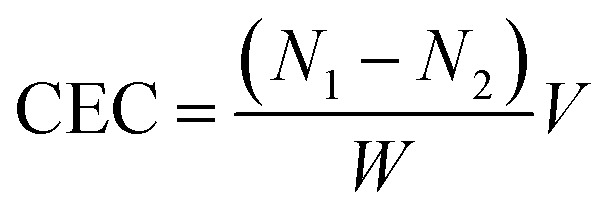
where CEC = cation exchange capacity (mmol g^−1^), *N*_1_ = initial concentration of NaOH solution (N), *N*_2_ = concentration of NaOH solution after 24 hours shaken (N), *V* = initial volume of NaOH solution (mL), and *W* = weight of the activated carbon (g).

### Adsorption capacity of 3-MCPD

**Fig. 1:**
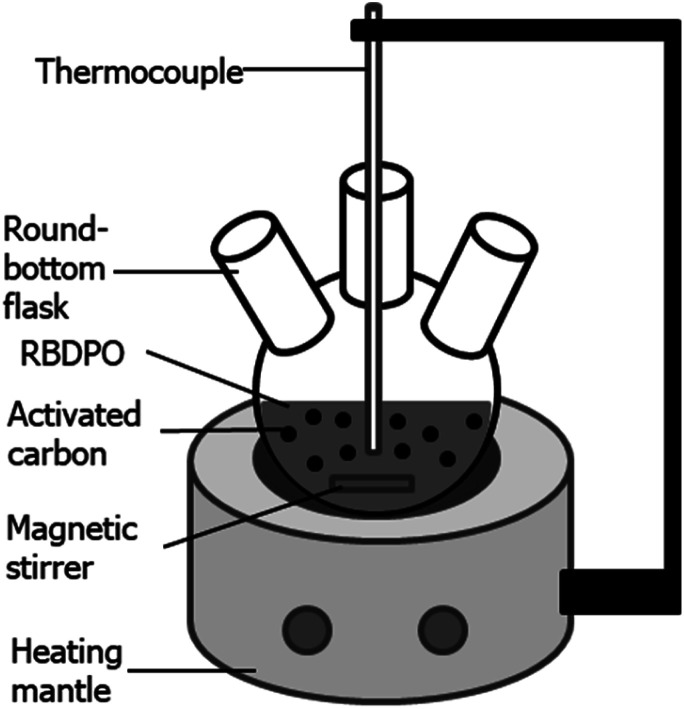
The scheme of instrument for the laboratory scale adsorption.

Activated carbon that was used as the adsorbent was chosen based on its preferable characteristics observed in previous experiments. Laboratory scale adsorption was conducted within a round-bottom flask (see Fig. 1). The solutions of 3-MCPD in methanol were prepared with various concentrations of 5, 10, 15 and 40 ppm. The adsorption was done at 40 °C for two hours using 2%-w of the activated carbon. Finally, the mixture was centrifuged at 5000 rpm and room temperature for 15 minutes. The solution was then prepared for GC-MS analysis.

### Adsorption of 3-MCPD from RBDPO

RBDPO was poured into the round-bottom flask and then heated to a certain temperature. After that, the activated carbon was added at a ratio of 2% w/w, and the adsorption was performed for 60 minutes. Finally, the RBDPO was separated with activated carbons by centrifugation (5000 rpm, room temperature, 15 minute). The adsorption of 3-MCPD from RBDPO was varied by the adsorption temperatures and the number of adsorption stages. The adsorption temperatures were varied from 35 up to 80 °C. On the other variation, the adsorption was conducted in single stage and quadruple stages of batch processes at constant adsorption temperature. The adsorbent was always changed for every single stage.

### The effect of adsorbent concentration on MCPD and GE removal from RBDPO

For this adsorption process, the concentration of activated carbon in RBDPO was varied by 1, 2, and 4%. The adsorption process was carried out with one-stage adsorption of batch process at temperature of 35 °C. After 2 hour adsorption, the sample was centrifuged at 5000 rpm and room temperature for 15 minutes. The samples were then prepared for GC-MS analysis.

### Analysis of 3-MCPDE and GE

The 3-MCPD in the RBDPO sample was analyzed by gas chromatography-mass spectrometry (GC-MS) with an indirect method. There were two assays in this analysis. The first assay was measurement of total 3-MCPD concentration in the sample. In this assay, the content of GE in RBDPO was converted into 3-MCPD ester (3-MCPDE). The second assay was measurement of the 3-MCPD concentration. About 100 mg sample was prepared in two vials which were labeled as Sample A and Sample B. Sample A was treated to convert GE into 3-MCPDE according to AOCS Cd 29a-13 with a little modification.^21^ Two milliliters of tetrahydrofuran and 30 μL of acidified NaCl solution (3 mg ml^−1^ NaCl in 5% H_2_SO_4_ solution) were added to vial A. The vial was then vortexed for 10 second and incubated at 50 °C for 15 minutes. After that, 3 ml of 0.6% NaHCO_3_ and 2 ml *n*-heptane were added to the vial A. The vial was then vortexed again for 10 seconds and the mixture was dried using N_2_ gas.

The dried contents gained in the vial A and the previous vial B were both treated according to a method developed by Federal Institute for Risk Assessment,^22^*i.e.*, method_82_FC-009-01 with a little modified. The sample then was then applied to GC-MS analysis. GC-MS analysis gave the total concentration of 3-MCPD from Assay A and the sole concentration of 3-MCPD from Assay B. Once the concentration values of Assay A and B gained, then the GE concentration can be calculated as written in eqn (2),^6^2
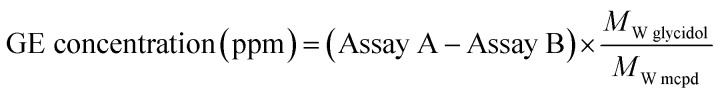
where, Assay A = total concentration of 3-MCPD in the sample A, Assay B = concentration of 3-MCPD in the sample B, *M*_W glycidol_ = molecular weight of glycidol (74.08 g mol^−1^), *M*_W mcpd_ = molecular weight of 3-monochoropropane-1,2-diol (110.54 g mol^−1^).

GC-MS analysis was done at Integrated Laboratory at Health Polytechnic of the Ministry of Health in Bandung, Indonesia. The GC-MS operating condition is shown at Table 1.

**Table 1:** GC-MS operating condition

Parameter	Condition
Brand	Agilent technologies
Type	GC 7890A-MS 5975
Oven temperature	60 °C for 1 minute
	6 °C min^−1^ to 90 °C for 1 minute
	20 °C min^−1^ to 280 °C for 5 minutes
Time	21.5 minutes
Injector temperature	175 °C
Injection method	Split less
Ionization mode	Electron impact
Carrier gas	Helium UHP
Gas flow	1.2 ml min^−1^
Injection volume	1 μL
SIM parameter	3-MCPD: 91, 147, 196
	3-MCPD-d5: 93, 150, 201
Column type	HP-5MS 5% phenyl methyl silox

### Equilibrium isotherm study of adsorption

The adsorption capacity of adsorbate at equilibrium condition could be calculated using eqn (3),^13^3
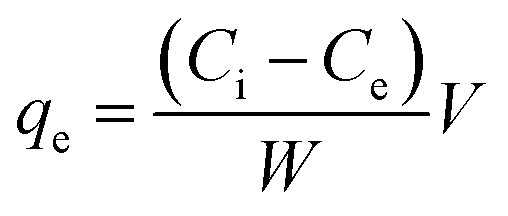
where *q*_e_ = adsorption capacity of adsorbate (mg g^−1^), *C*_i_ = initial concentration of adsorbate (mg L^−1^), *C*_e_ = concentration of adsorbate at equilibrium (mg L^−1^), *V* = initial volume of adsorbate solution (L), *W* = weight of the activated carbon (g).

In this study, five adsorption isotherm models were fitted to the data. Those models were Langmuir, Freundlich, Dubinin–Raduskevich, Temkin, and Flory–Huggins. The Langmuir isotherm model is written in eqn (4),^23^4
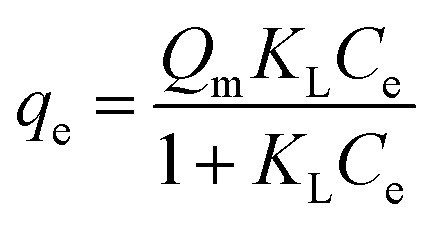
where *K*_L_ = Langmuir constant related to energy of adsorption capacity (L mg^−1^) and *Q*_m_ = maximum adsorption capacity (mg g^−1^). The linear form of Freundlich isotherm model is written in eqn (5),^23^

5 *q*_e_ = *K*_f_*C*_e_^1/*n*^

where *K*_f_ = adsorption capacity at unit concentration (mg L^−1^) and 1/*n* = adsorption intensity. Dubinin–Raduskevich isotherm model is represented in eqn (6)–(8),^23^

6 *q*_e_ = *q*_m_ exp(−*K*_DR_*ε*^2^)

7
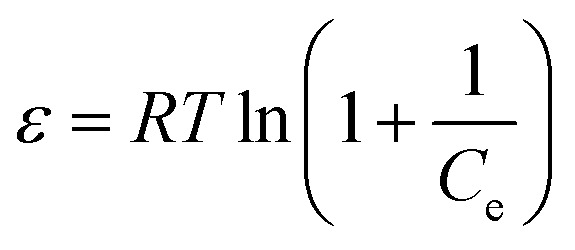
8
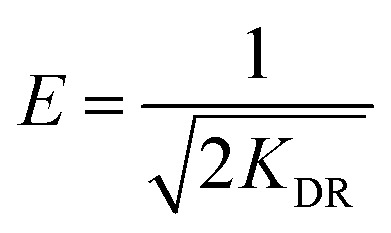
where *K*_DR_ = a constant corelated with free energy of adsorption, *q*_m_ = adsorption saturation capacity. *ε* = polanyi potential that calculated from eqn (7), *R* = ideal gas constant (8.314 J mol^−1^ K^−1^), and *T* = temperature (K). Free energy of adsorption can be calculated using eqn (8).

Temkin isotherm model is represented in eqn (9),^23^9
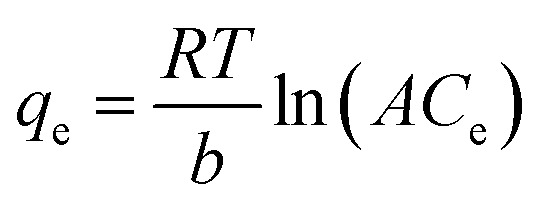
where *b* = the Temkin constant related to heat sorption (J mg^−1^). *A* is Temkin isotherm constant (L g^−1^). Flory–Huggins isotherm model is shown in eqn (10),^23^10
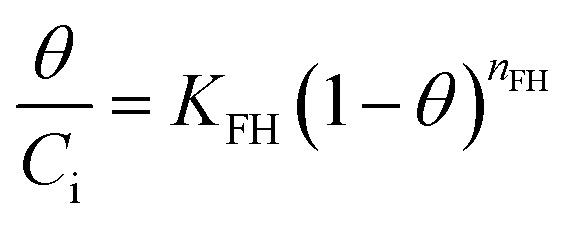
11
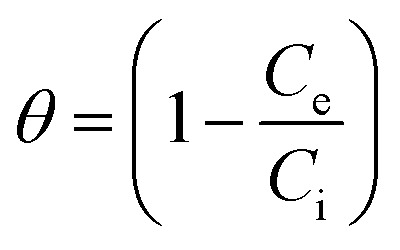
12
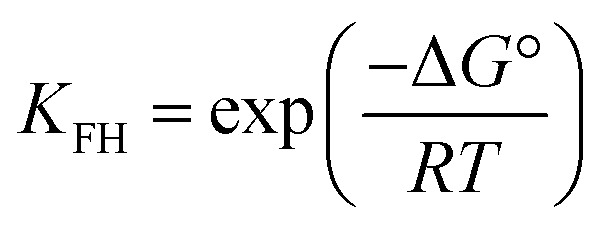
where *K*_FH_ is equilibrium constant (L mol^−1^) and *n*_FH_ is number of adsorbates occupying the adsorption sites. The *θ* is defined in eqn (11). The value of *K*_FH_ is used to calculate Δ*G*° of the adsorption using eqn (12). All those models were fitted to the data nonlinearly using curve fitting toolbox in MATLAB R2016a software.^24^

## Results and discussion

### Characterizations of activated carbon

**Fig. 2:**
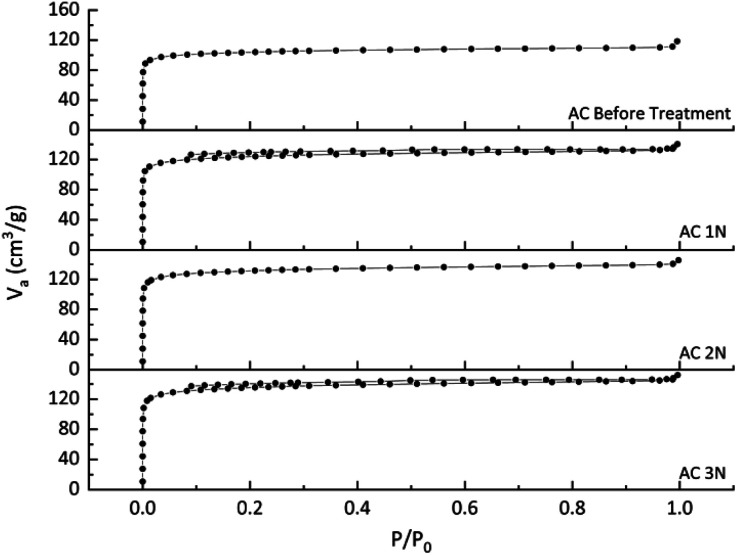
Isotherm curve of activated carbons before and after treatment.

N_2_ physisorption analyser using the BET method was used to identify the adsorbent adsorption isotherm and measure the specific surface area and pores volume. The isotherm curve is shown in Fig. 2. Fig. 2 shows that all activated carbons before and after treatment were isotherm type I. Isotherm type I indicates that the adsorbent was typically microporous, with the exposed surface locating almost completely inside the micropores, which left little or no external surface for further adsorption once filled with adsorbate.^25^ The type I is concave to *P*/*P*_0_ axis, and the amount adsorbed reaches a limiting value (*P*/*P*_0_ → 1). *P*/*P*_0_ is the relative pressure, which is the ratio of the adsorbate gas equilibrium pressure to its saturated equilibrium vapor pressure.^25^ It is obviously depicted in Fig. 2 that acid treatment can increase the volume of N_2_ adsorbed which indicates an increased pore volume.

The surface area and pore distribution of activated carbon before and after treatment is shown on Table 2. The surface area of activated carbon before the treatment was 404 m^2^ g^−1^, lower than the activated carbon after the treatment. The acid treatment on activated carbon resulted in the inorganic compound removal,^26^ so that the surface area and pore volume of the activated carbon treated with HCl were increased. The micropore and mesopore volumes of the pre-treated activated carbon were increased. It can be obviously seen that the acid treatment on activated carbon enhanced the micropore and mesopore distributions. The percentage of micropore (more than 50%) in Table 2 corresponds to Fig. 2 which showed the type I of isotherm model (typically microporous). The increasing surface area was influenced by the concentration of HCl used. The total pore volume of the pre-treated activated carbon was also influenced by the concentration of HCl, but the total pore volume of AC 2 N was slightly lower than that of AC 1 N. However, the concentration of HCl did not influence the ratio of activated carbon's pore size significantly.

**Table 2:** Specific surface area and pore volume of the activated carbons before and after treatment

Adsorbents	*S*_BET_ [m^2^ g^−1^]	*V*_micro_ × 10^3^ [cm^3^ g^−1^] (fraction)	*V*_meso_ × 10^3^ [cm^3^ g^−1^] (fraction)	*V*_macro_ × 10^3^ [cm^3^ g^−1^] (fraction)	*V*_total_ × 10^3^ [cm^3^ g^−1^]
AC_Before treatment_	404	33.69 (57%)	16.85 (28%)	5.05 (9%)	59.34
AC 1 N	486	40.72 (56%)	21.96 (30%)	5.41 (7%)	72.68
AC 2 N	518	40.50 (58%)	21.16 (30%)	3.29 (5%)	69.46
AC 3 N	532	43.70 (57%)	24.02 (31%)	4.65 (6%)	77.31

**Fig. 3:**
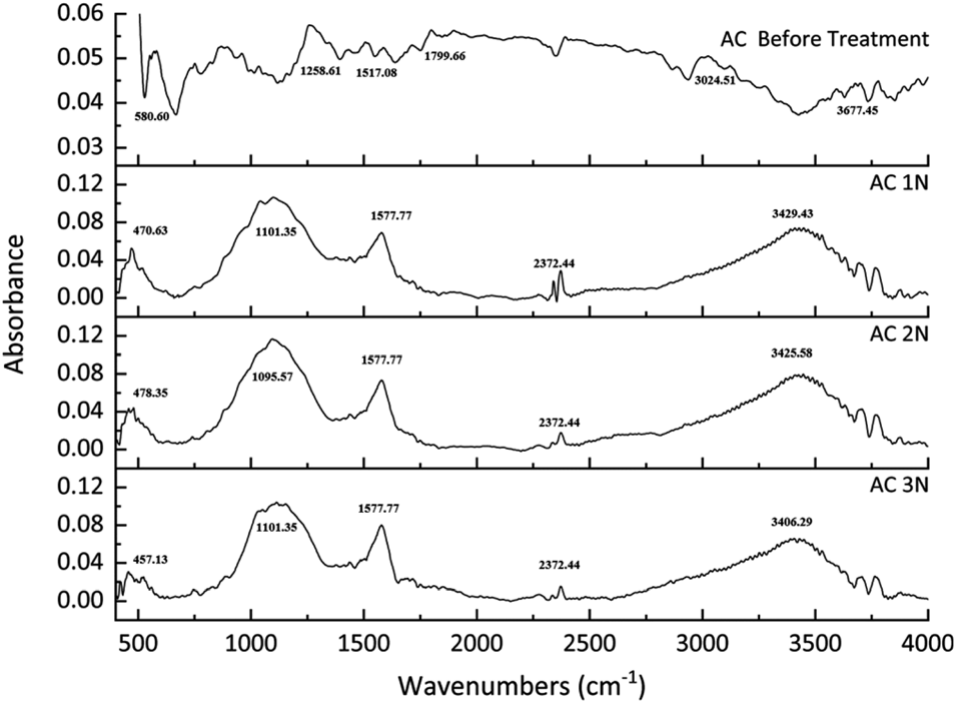
FTIR results of the activated carbons before and after treatment.

The FTIR spectroscopy was used to identify the chemical groups on the activated carbon's surface before and after treatments. The results can be seen in Fig. 3. The functional group of activated carbon before treatment was slightly different with activated carbon after treatment. For untreated activated carbon, the peak appears at approximately 550 cm^−1^ resulting from C–C stretching. Peaks at approximately 1258 cm^−1^ can be assigned to C–O stretching from acids, esters, or ethers group.^27^ Other peaks appear at approximately 1517 cm^−1^ and 1799 cm^−1^ ascribed from C=O stretching from carboxyl.^28^ For treated activated carbon, FTIR spectrum for every activated carbon after treatment did not show any significant difference. The broad peak of treated activated carbon at approximately 3421 cm^−1^ belongs to an O–H bond, which indicates hydroxyl and carboxyl groups.^29^ Peak appearing at 1579 cm^−1^ is attributed to C=C bond of aromatic ring.^30^ Another broad and strong peak at 1091 cm^−1^ belongs to C–O bond,^31,32^ which is in lactone group.^33^

From the FTIR spectrum peaks, many oxygen-containing chemical groups appeared in the treated activated carbon. Acid-washing the activated carbon can increase those chemical groups by oxidizing the surface of the activated carbon.^29^ Moreover, acid treatment could significantly increase the hydroxyl and carboxyl groups of the untreated activated carbon.^33^

The concentration of lactone increased with the increase of acid concentration from 1 N to 2 N, but it decreased at 3 N. On the other hand, the concentration of carboxylic compound increased with the increase of the acid concentration. The modified activated carbon resulted in a change in acidity which is related to the amount and types of functional groups on the surface of the activated carbon. The decrease of pH_PZC_ value and the total carbon acidity indicated a high concentration of acid sites.^33^

Adsorption process by using activated carbon involves interaction between adsorbate and carbon surface either through electrostatic or non-electrostatic including van der Waals forces, hydrogen bonding or hydrophobic interaction.^34^ XRF analysis was conducted on untreated and treated activated carbon (see Table 3), to determine the composition of inorganic elementals. According to XRF analysis carried out on untreated activated carbon, *K* was the highest content, and was followed with Ca, Si, S, and P. Meanwhile, XRF analysis of activated carbon treated with 2 N HCl showed that Cl was the highest content, which resulted from 24 hours acid-washed by HCl solution, and also hydrogen bonding between Cl^−^ and the surface of activated carbon, although the bonding was poor.^35–37^ After Cl content, the chemical composition in the activated carbon followed with Si, S, K, Ca, and Fe. Based on the XRF analysis, heat treatment and acid wash could reduce ash content (Ca, K, and P) of activated carbon, which were impurities of the activated carbon. The Fe, S, and Si was slightly increased, possibly from impurities of acid solution and washing water of acid wash treatment, in the form of Fe^2+^, Fe^3+^, SO_4_^2−^, and SiO_2_.^38^ In this analysis, carbon elemental was not detected before and after treatment using XRF analysis, because XRF analysis can only measure inorganic elements. However, the carbon organic was detected by CHN Analyzer as discussed in Material and Methods section. The chemical composition detected on this activated carbon is slightly similar to the activated carbon used by Dittmann *et al.* (2020).^39^ The Si content in the activated carbon depends on the Si content in the original biomass of the activated carbon.^40^

**Table 3:** X-ray fluorescence (XRF) measurements of activated carbons before and after treatment

Element	AC_Before treatment_ (%-w)	AC 2 N (%-w)
Si	10.11	12.95
P	1.21	n/a
S	2.75	6.58
K	71.24	3.81
Ca	14.69	3.09
Fe	n/a	0.67
Cl	n/a	72.49

### Activated carbon's pH_PZC_ and CEC

The pH_PZC_ value represent the surface charge of carbon in solution and also exhibit the acidity/alkalinity of the adsorbent. The pH_PZC_ and CEC value of the treated activated carbon are shown in Table 4. The value of pH_PZC_ means that the activated carbon surface charge at that pH value is zero. In this research, the value of pH_PZC_ of the treated activated carbon was slightly lower than the untreated activated carbon. This shows that the surface of activated carbon tends to basicity before treated using acid, while activate carbon is more neutral after treatment. This can be explained because the surface of the activated carbon contains many functional groups such as carboxylate and phenolic groups.^41^ This is in line with the results of research conducted by Beker *et al.* (2010), which states that the overall surface charge of activated carbon becomes positive at low pH conditions, whereas the overall surface charge of activated carbon becomes negative at high pH conditions.^41^ The value of pH_PZC_ of the treated activated carbons did not differ significantly. The value range of pH_PZC_ was from 6.85 to 7.33 which means that the surface charge of the activated carbon's surface is zero at those pH range. The values of pH_PZC_ also indicate that the surface of the treated activated had balanced content between acidic groups and basic groups.^6,42^

**Table 4:** Values of pH_PZC_ and CEC of the activated carbons before and after treatment

Adsorbents	pH_PZC_	CEC (mmol g^−1^)
AC_Before treatment_	7.65	1.0
AC 1 N	6.85	0.9
AC 2 N	7.3	1.8
AC 3 N	6.87	1.4

From the measurement of the CEC (Table 4), the acid wash treatment increased the CEC of activated carbon, except activated carbon treated with the HCl 1 N, possibly because the acid concentration was low. Activated carbon treated with HCl 2 N had the highest value of CEC, which was 1.8 mmol g^−1^. The CEC of activated carbon is relatively low.^43^ However, that value was near the average of activated carbon CEC, which is 1.63 mmol g^−1^.^20,44–46^

### Equilibrium studies of the 3-MCPD adsorption using treated activated carbon

The adsorption of MCPD compounds was due to esterification of carboxyl groups on the activated carbon surface with the chloride site of 3-MCPD,^47^ not the hydroxyl site or ester site. Therefore, the isotherm model was measured with the adsorption of 3-MCPD solution as adsorbate solution.

**Fig. 4:**
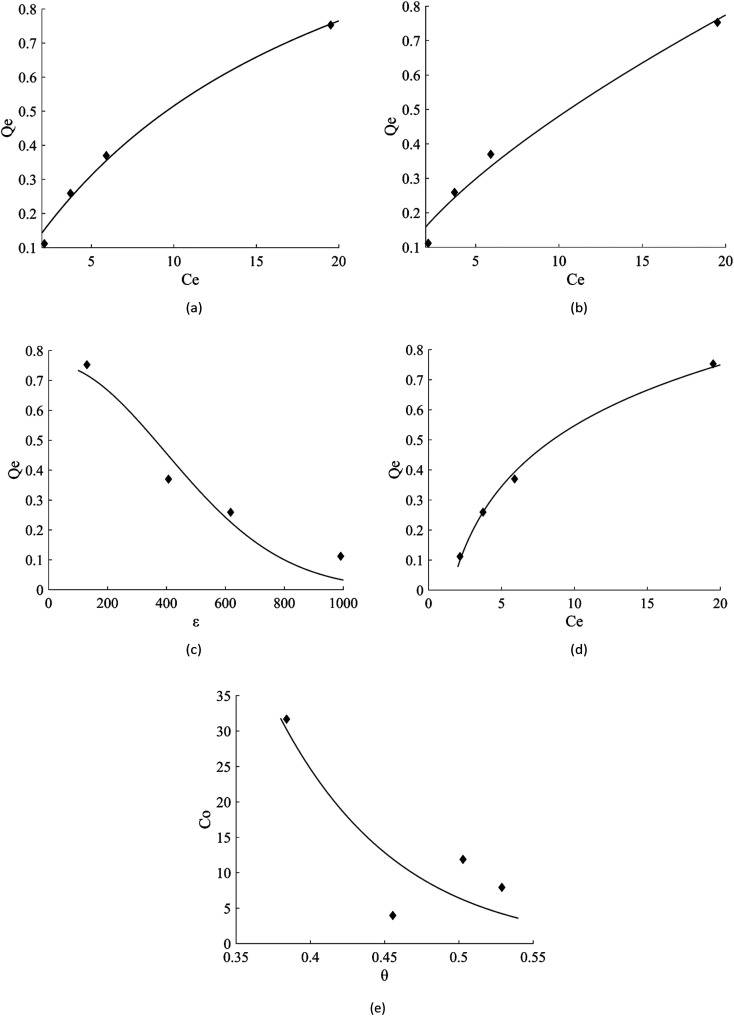
Isotherm model plots of 3-MCPD adsorption, (a) Langmuir, (b) Freundlich, (c) Dubinin–Raduskevich, (d) Temkin, (e) Flory–Huggins.

The linear regression plot of every isotherm models can be seen at Fig. 4 and their parameter values are shown at Table 5. From the results, Langmuir, Freundlich, Dubinin–Raduskevich, and Temkin isotherm model had the high *R*^2^ values and Temkin model was the highest, hence the most fit model for the adsorption of 3-MCPD. From Langmuir isotherm model, the maximum adsorption capacity was 1.48 mg g^−1^, different from the value from Dubinin-Raduskevich model which was 0.60 mg g^−1^. The activation energy of 3-MCPD adsorption from the same model was 0.4 kJ mol^−1^. From Flory–Huggins isotherm model, the Δ*G*° of the 3-MCPD adsorption was −14.13 kJ mol^−1^, which means the adsorption process is spontaneous. Temkin isotherm model can be used to approximate the heat of 3-MCPD adsorption.^23^ From the results, the *K*_T_ and *b* were 0.65 L mol^−1^ and 8914 J mol^−1^, respectively. Because the Temkin constant is related to heat of adsorption, it could be assumed that the heat of adsorption (Δ*H*) of 3-MCPD was −8914 J mol^−1^ (negative punctuation because the adsorption is exothermic). According to Desiraju and Steiner (1999), and Jeffrey and Saenger (1991), the bonding energy of adsorption in this research was weak hydrogen bond.^36,37^

**Table 5:** Values of each parameter isotherm model for the adsorption of 3-MCPD

Isotherm model	Parameters				
Langmuir	*Q*_m_ (mg g^−1^)	*K*_L_ (L mg^−1^)		*R* ^2^	RMSE
	1.48	0.05		0.9860	0.0324
Freundlich	*n*	*K*_f_ (mg g^−1^)		*R* ^2^	RMSE
	1.45	0.10		0.9692	0.0481
Dubinin–Raduskevich	*Q*_m_ (mg g^−1^)	*K*_DR_ (mol^2^ J^−2^)	*E* (J mol^−1^)	*R* ^2^	RMSE
	0.60	3.17 10^−6^	397.339	0.9354	0.0854
Temkin	*β*	*b* (J mol^−1^)	*A* (L mol^−1^)	*R* ^2^	RMSE
	0.29	8914	0.651	0.9963	0.0204
Flory–Huggins	*n*	*K*_FH_ (L g^−1^)	Δ*G*° (kJ mol^−1^)	*R* ^2^	RMSE
	6.15	228.5	−14.134	0.6307	7.482

### The effects of adsorption temperature and number of stages of adsorption on the 3-MCPDE removal

In this investigation, the adsorbent used was activated carbon which had been treated with 2 N HCl (AC 2 N) because this adsorbent exhibited the highest CEC value. AC 2 N adsorbent exhibited slightly lower surface area and pore volume than AC 3 N adsorbent. However, AC 2 N adsorbent was better chosen as an adsorbent because its production process requires less HCl than the production process of AC 3 N. This will have an impact on operating costs when the process is scaled up.

Concentration of 3-MCPDE in RBDPO was measured as total 3-monochloropropane-1,2-diol (3-MCPD) concentration in RBDPO. A measurement of 3-MCPDE cannot be taken directly with GC-MS; it needs to be in a non-esterified form to determine. Although 3-MCPD is also contained in RBDPO, its concentration is much lower than the concentration of 3-MCPDE.^48^

**Fig. 5:**
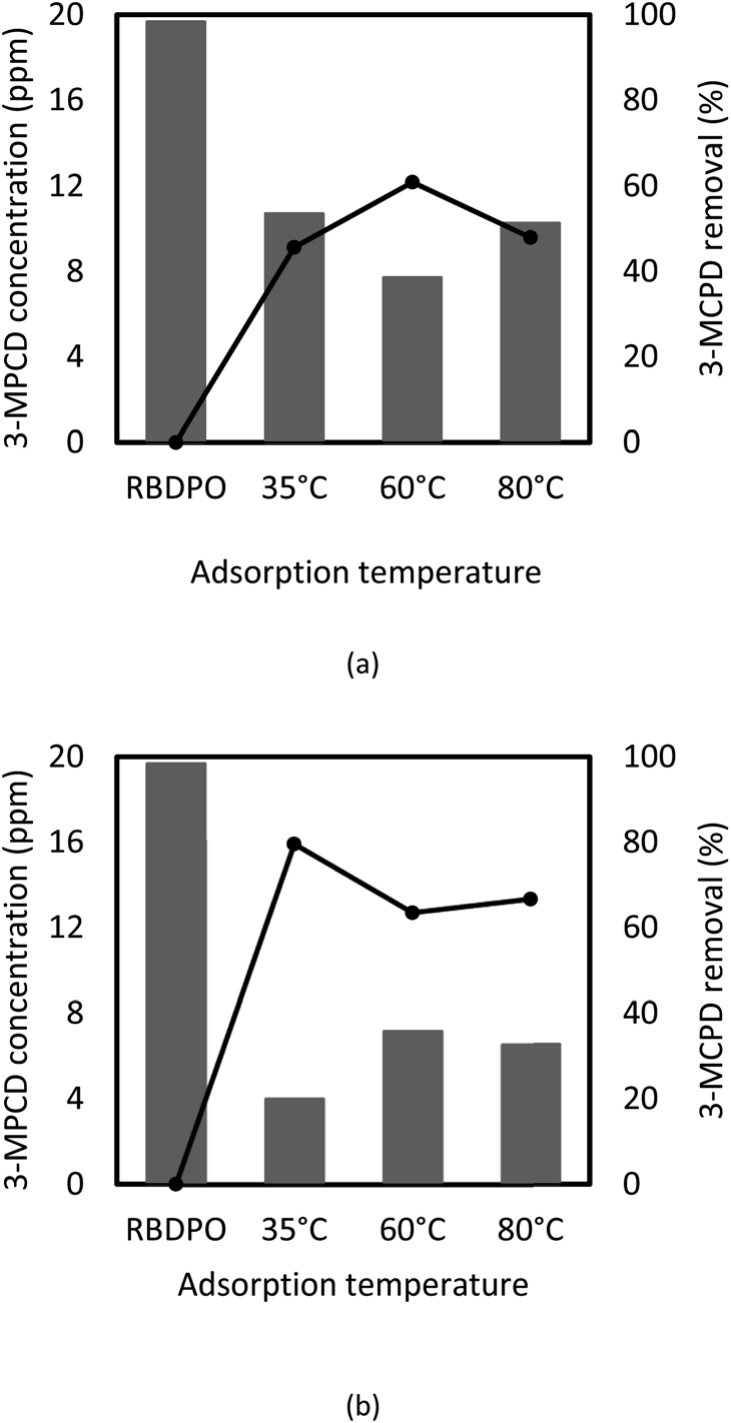
Concentration of 3-MCPD (bar chart) and percent removal of 3-MCPD from RBDPO (circle with line) before and after adsorption at temperature of 80, 60, and 35 °C in (a) single stage of batch process, and (b) quadruple stages of batch process.

GC-MS was used to analyze the sample before and after adsorption, and the results are shown in Fig. 5. The obtained RBDPO sample contained 19.67 ppm of 3-MCPD. According to Figure 5, 3-MCPD concentration in RBDPO after the adsorption processes using the treated activated carbon was reduced, which showed that the pre-treated activated could remove the 3-MCPDE in RBDPO.

The reductions of 3-MCPD concentrations were varied based on the process temperature and the stepwise stages of the process (see Fig. 5). At process temperature at a process temperature of 35 °C and four stages of the batch process, 3-MCPDE removal was the highest at 80%, which was better than the previous research showed.^7^ It indicates that lower temperature gives better reduction of 3-MCPDE concentration, and this corresponds to Cheng's research,^6^ which showed more efficient removal at the lower temperature. The batch stepwise process also seems to increase the removal efficiency of 3-MPCDE. However, at the temperature of 60 °C, there only was a little change in the 3-MCPD concentration in RBDPO between 1- and 4-stages the adsorption process. The results indicated that the lower the temperature and the greater the number of batch adsorption stages resulted in higher 3-MCPD removal.

### Effect of adsorbent concentration on 3-MCPD dan GE removal

**Fig. 6:**
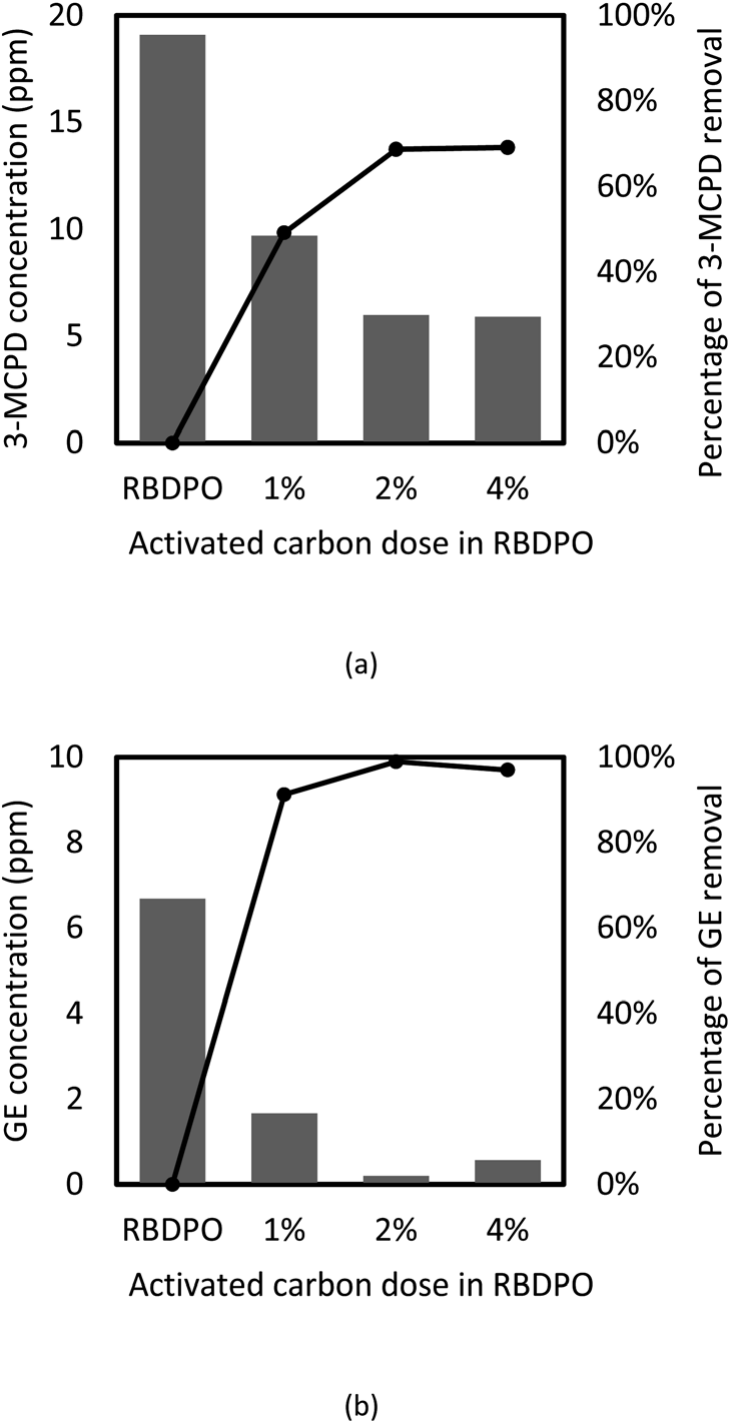
The concentration (bar chart) and the percentage of removal (circle with line) of (a) 3-MCPD and (b) GE from RBDPO before and after adsorption with various activated carbon dose at *T* = 35 °C.

The effects of adsorbent concentration on 3-MCPD and GE removal were investigated, and the results are given in Fig. 6(a) and (b). The initial concentration of 3-MCPD and GE were 19.7 ppm and 6.7 ppm, respectively. The concentration decreases and percentage of removal of 3-MCPD and GE from RBDPO before and after adsorption using various concentration of activated carbon are depicted in Fig. 6(a) and (b), respectively.

The initial GE concentration in RBDPO was 6.68 ppm and after the adsorption process using activated carbon, the final concentration could reach 0.2 ppm. The highest GE removal (97%) was obtained when 2%-w activated carbon was applied as adsorbent. The adsorption capacity of GE by the activated carbon was detected at 29.68 mg g^−1^. Activated carbon generally has a hydrophobic surface so it is good for adsorbing non-polar molecules, such as GE.^49^ The similar result on GE removal was also obtained by Cheng *et al.* (2017)^6^ that the adsorption GE from palm oil using acid-washed activated carbon could reach up to 95.5% at *T* = 35 °C. The results of GE removal in this study were slightly higher than those obtained by Cheng *et al.* (2017). The different can occur when the activated carbon pore structures, such as surface area and pore volumes, are different.

In this study, the activated carbon was used not only for adsorbing GE, but also for MCPD compounds (3-MCPD and 3-MCPDE) removal from RBDPO. About 68% of the total MCPD compounds were removed from RBDPO (see Fig. 6). Referring to the adsorption percentage, it is clear that the activated carbon can adsorb GE better than total MCPD compounds. The activated carbon had better adsorption to GE than 3-MCPDE because of the molecules size. The 3-MCPD molecule contains two hydroxyl groups that can be esterified to form 3-MCPD monoester of 3-MCPD diester. However, the glycidol molecule only has one hydroxyl group so it just can be a glycidyl monoester.^3^ Meanwhile, in the palm oil, nearly 90% of 3-MPCDE is in diester form.^50^ Because it has two esters, 3-MCPD diester should have higher molecular size than 3-MCPD monoester and glycidyl ester. Smaller molecules are easier to enter activated carbon's pore, therefore the activated carbon was easier to adsorb GE than 3-MCPDE.

The ability of the activated carbon to adsorb non-polar compounds (3-MCPDE and GE) or polar compounds (3-MCPD and glycidol) is also related to its hydrophobicity/hydrophilicity properties. The hydrophobic/hydrophilic properties of activated carbon are related to its oxygen content. In general, the activated carbon was indicated as a nonpolar adsorbent, so it is possible to adsorb 3-MCPDE and GE which are nonpolar molecules. This results in a strong affinity between the nonpolar molecules and the activated carbon. However, the activated carbon, which exhibits oxygen, will have a polar site.^51,52^ Therefore, the activated carbon has the ability to adsorb polar compounds such as 2-MCPD, 3-MCPD, and glycidol (non-esterified form). Previous research has proven that polar compounds such as water can bond to activated carbon *via* hydrogen bonding followed by additional water molecule groupings at this site.^51,52^ The activated carbon modified by acid treatment can induce the surface to become more polar,^53^ and also resulting the adsorbent more selective to adsorb 3-MCPDE, GE, and its non-esterified form which was more polar than triglyceride. In conclusion, the activated carbon is mostly nonpolar, but it also has some polar sites.

The activated carbon can also bind the adsorbates *via* London dispersion forces that allows to adsorb larger molecules and non-polar molecules. The London dispersion force is a temporary attractive force that occurs when the electrons in two adjacent atoms occupy positions, so the atoms make temporary dipoles to synchronize the distribution of electron.^54^ The London dispersion force occurs in the non-polar molecules, such as 3-MCPDE and GE. The adsorption of organic matter to activated carbon is influenced by several factors, such as characteristics of organic molecules (polarity, functional groups), characteristics of activated carbon (surface area, pore size distribution and functional groups on the surface), and operating conditions (temperature, pressure, time, adsorbent dose).^55,56^ Moreover, based on research conducted by Li *et al.* (2012), the adsorption process of organic pollutant by using activated carbon particularly occurs *via* physical adsorption through van der Waals forces between activated carbon and adsorbate.^57^

## Conclusions

Activated carbon has been modified using acid. Acid treatment using 2 N HCl on activated carbon gave a good pore volume distribution. It was successfully demonstrated that the activated carbon treated with 2 N HCl could adsorb both 3-MCPD and GE. By fitting the equilibrium data using various isotherm models, the maximum adsorption capacity, activation energy, and Gibbs free energy can be estimated, which are 1.484 mg g^−1^, 0.4 kJ mol^−1^, and −14.134 kJ mol^−1^, respectively. The low adsorption temperature and the large number of batch stages can increase the percentage of 3-MCPD removal in RBDPO. The best operating conditions to reduce by 80% of the initial concentration of 3-MCPD were at a temperature of 35 °C and the operation was carried out in quadruple batch stages carried out in series. On the other hand, the activated carbon can remove about 97% GE concentration in RBDPO. The activated carbon showed better adsorption of non-polar compounds than adsorption of polar compounds. The removal of GE content in RBDPO was better than the removal of 3-MCPD using activated carbon. The determined adsorption capacity of GE was 29.68 mg g^−1^. The highest percentage of GE removal was 97% at 35 °C operating temperature.

## Author contributions

The authors have participated in this manuscript preparation.

Elvi Restiawaty: conception, critical revising for important intellectual content, data analysis and interpretation, drafting, and approval of the final version; Aulia Maulana, Christian Aslan, Neng Tresna Umi Culsum: execute and investigate the experiment, drafting, data analysis and interpretation; Veinardi Suendo, Norikazu Nishiyama, Yogi Wibisono Budhi: critical revising for important intellectual content.

## Conflicts of interest

There are no conflicts to declare.

## Supplementary Material
